# Rare Case of Female with Pelizaeus Mertzbacher Disease due to deletion of Proteolipid Protein 1: A Case Report

**DOI:** 10.31729/jnma.3824

**Published:** 2018-12-31

**Authors:** Masanosuke Kinoshita, William Roston

**Affiliations:** 1 Department of Family Medicine, Theodosia Family Medical Clinic, Theodosia, USA

**Keywords:** *deletion*, *female*, *Genetic testing*, *Pelizaeus Merzbacher Disease*, *Proteolipid Protein 1*

## Abstract

Pelizaeus Merzbacher Disease is a rare X-linked central nervous system disease involving the proteolipid protein 1 gene. Patients exhibit signs for instance nystagmus, hypotonia, ataxia. We report a three-year-old female patient with chief compliant of developmental delay. On physical examination, patient was alert but had poor eye contact while sitting in a stroller. Since no chromosomal evaluation was performed, a chromosomal microarray testing was performed. Review of geneticist report indicated that patient carries a deletion of at least 2.26 Mb within cytogenetic band Xq22.1 to Xq22.2 which is known to contain 39 genes. Out of the 39 genes, proteolipid protein 1 is associated with known clinical disorder; Pelizaeus Merzbacher Disease. Our case highlights the second only known female with Pelizaeus Merzbacher Disease due to deletions of proteolipid protein 1 gene. For a patient with developmental delay, the importance of performing genetic testing and/or radiological imaging early on is strongly recommended.

## INTRODUCTION

Pelizaeus Merzbacher Disease (PMD) is a rare X-linked central nervous system (CNS) disease involving the proteolipid protein 1 (PLP1) gene on Xq22.^1^ PLP1 gene codes for myelin protein which leads to hypomyelination.^1^ PLP1 gene is one of leukodystrophies characterized by degeneration of white matter in CNS.^1^ Patients exhibit signs that progressively worsen for instance nystagmus, hypotonia, ataxia, convulsions, muscle contractions, language delay.^2,3^ PMD affects males more than females. There are very few reported cases where females are affected, but our case is the second only reported female patient with PMD due to a deletion of PLP1 gene.^1^

## CASE REPORT

**Figure 1.**
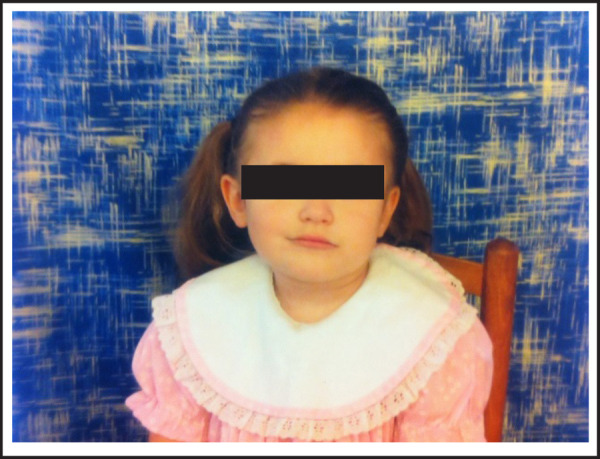
Photograph of the patient at the age of three.

A three-year-old female patient was accompanied by her mother in a stroller with chief compliant of developmental delay (Figure 1). Patient was naturally conceived and prenatal ultrasound was normal. Patient was spontaneously vaginally delivered at 30 weeks. Patient passed the newborn vision screen and hearing screen, but she was admitted to Neonatal Intensive Care Unit (NICU) due to incomplete lung formation and difficulty with feeding as well as hydration. Patient was on Nasal Gastric (NG) feeding tube but began bottle feeding two weeks prior to discharge. Patient's mother describes her daughter to have very delayed milestones since birth. Patient did not start to roll over or sit on her own until two years old. Patient began to crawl at three years old. Patient is unable to communicate, recognize and identify common objects and pictures, walk or climb upstairs, complete simple maneuvers, for instance turn rotating handles, show wide range of emotions such as being sad, angry, and happy. Patient's father is alive and does not show any signs of neurological degeneration at age 50. Similarly, patient's mother does not show any signs of neurological degeneration at age 34. At present, patient is undergoing occupational and speech therapy. The goals for occupational therapy for patient are to strengthen her upper and lower extremity muscles. The goals for speech therapy are to improve her swallowing on oral intake.

On physical examination, patient was alert but had poor eye contact while sitting in a stroller. Patient had broad forehead with small pointed nose. Patient's left eye had strabismus but otherwise normal pupil and conjunctiva. Right eye had normal pupil and conjunctiva. There was no nystagmus and coloboma. Patient had thin upper lip with mild dental decay. She did make clicking noises with her tongue. Cardiovascular exam of the heart revealed regular rate and rhythm. Auscultation of the lung was clear. Patient's abdomen was soft and non-distended. Patient was able to hold her head up, but otherwise she preferred to look down to the ground. Patient did not have a pincer grasp. She also had a difficult time grasping objects with her hands. Upper extremity strength appeared to be weak although there was no muscle atrophy. Lower extremity strength was also weak as patient was unable to bear weight on her legs. Patient preferred to sit. While sitting in a stroller, patient had her legs spread out at the hip joints. There were no signs of hip dysplasia. Since no chromosomal evaluation was performed, it was decided to have a geneticist specialist assist in managing patient's symptoms. At this point, the primary differential diagnosis was global developmental delay.

At the geneticist clinic, patient was evaluated and a chromosomal microarray testing was performed. From the chromosomal microarray testing result, patient was diagnosed to have PMD. Review of geneticist report indicated that patient carries a deletion of at least 2.26 Mb of a region within cytogenetic band Xq22.1 to Xq22.2 and a duplication of at least 523 kb of a region within cytogenetic band 22q11.23. The deletion in Xq22.1q22.2 is known to contain 39 genes.

Out of the 39 genes, PLP1 is associated with known clinical disorder: PMD. Patient's mother was advised in continue having the patient attend occupational and speech therapy while coordinated care with geneticist in managing patient's current symptoms was decided.

## DISCUSSION

To begin, our case highlights a rare case of PMD in a female patient. Our patient is also the second only known female with PMD due to deletions of PLP1 gene.^1^ PMD is an X-linked neurodegenerative leukodystrophy disorder caused by mutation of PLP1 gene. PLP1 mutation can be due to point mutation, duplication, or deletion.^1–3^ PLP1 mutation leads to hypomyelination leading to reduced neurological function. As an X-linked disease, PMD is often seen in males and rarely observed in females. For a female patient to be affected, X-linked recessive inheritance pattern by gene mutation and skewed X inactivation needs to take place.^1,2^ Patient's mother most likely then has the affected X-linked gene which patient obtained it in an X-linked recessive inheritance with a skewed X inactivation. A review of literature indicates that there are currently at least 13 other cases of affected females with a genetic diagnosis of PMD.^1–4^ Out of the thirteen cases examined, our patient is the second only known female with deletions of PLP1 gene.^1^ Twelve other female cases were due to point mutations or duplications of the PLP1 gene.^1–4^

In addition, the second highlight of our case is the importance of performing genetic testing and/ or radiological imaging early on for a patient with developmental delay. Our patient was diagnosed with PMD at 3 years old due to genetic testing. This early diagnosis was made possible due to patient's mother seeking immediate medical care for her daughter after she showed delayed milestone achievement. Some of the known early classical PMD signs and symptoms are hypotonia, nystagmus, and ataxia.^2,3^ Additional PMD signs and symptoms that appear with age are delayed motor skills development, spasticity, stridor, dysarthria, titubation, dystonia, choreiform, and seizures.^2,3^ However, not all patients will show these signs and symptoms, and they may present as a milder unnoticeable form even if present. Through genetic testing, PMD diagnosis can be made.^1–3^ Despite the significant role genetic testing can provide in medical diagnosis, it is expensive and not many insurance companies will authorize this service. A review of literature indicates that PMD can be diagnosed by magnetic resonance imaging (MRI) as unique features to PMD are distinctively observed on imaging.^5^ A case of female with PMD was diagnosed alone by radiological imaging.^4^ Through MRI of the brain when there is 1. diffuse hypomyelination, 2. increased signal intensity on T2 weighted for fluid attenuated inversion recovery (FLAIR) scans in white matter region, 3. corpus callosum thinning, and 4) cerebral hemisphere atrophy, it is characteristic of PMD.^5^ In order to diagnose PMD, radiological imaging of the brain is then recommended to be performed as an alternative to genetic testing.

Our patient is the second only female affected with PMD due to gene deletion. Females with PMD is very rare, and gene deletion of PLP1 leading to PMD in females is likewise rare. PMD needs to be considered as one of the differential diagnosis in a female patient with reduced neurological function. PMD is best diagnosed by genetic testing as in our patient's case. However if genetic testing is not possible, radiological imaging of the brain is recommended where hypomyelination, increased signal intensity in white matter region, corpus callosum thinning, and cerebral hemisphere atrophy are characteristic of PMD.
